# How does tautomerization affect the excited-state dynamics of an amino acid-derivatized corrole?

**DOI:** 10.1007/s11120-021-00824-4

**Published:** 2021-03-12

**Authors:** John A. Clark, Rafał Orłowski, James B. Derr, Eli M. Espinoza, Daniel T. Gryko, Valentine I. Vullev

**Affiliations:** 1 Department of Bioengineering, University of California, Riverside, CA 92521 USA; 2 Institute of Organic Chemistry, Polish Academy of Sciences, Kasprzaka 44/52, 01-224 Warsaw, Poland; 3 Department of Biochemistry, University of California, Riverside, CA 92521 USA; 4 Department of Chemistry, University of California, Riverside, CA 92521 USA; 5 Materials Science and Engineering Program, University of California, Riverside, CA 92521 USA; 6 Present Address: College of Bioengineering, University of California, Berkeley, CA 94720 USA

**Keywords:** Porphyrinoids, Corroles, Tautomers, Hydrogen bonding, Kinetic isotope effect, Transient-absorption spectroscopy

## Abstract

In the first two decades of the XXI century, corroles have emerged as an important class of porphyrinoids for photonics and biomedical photonics. In comparison with porphyrins, corroles have lower molecular symmetry and higher electron density, which leads to uniquely complementary properties. In macrocycles of free-base corroles, for example, three protons are distributed among four pyrrole nitrogens. It results in distinct tautomers that have different thermodynamic energies. Herein, we focus on the excited-state dynamics of a corrole modified with l-phenylalanine. The tautomerization in the singlet-excited state occurs in the timescales of about 10–100 picoseconds and exhibits substantial kinetic isotope effects. It, however, does not discernably affect nanosecond deactivation of the photoexcited corrole and its basic photophysics. Nevertheless, this excited-state tautomerization dynamics can strongly affect photoinduced processes with comparable or shorter timescales, considering the 100-meV energy differences between the tautomers in the excited state. The effects on the kinetics of charge transfer and energy transfer, initiated prior to reaching the equilibrium thermalization of the excited-state tautomer population, can be indeed substantial. Such considerations are crucially important in the design of systems for artificial photosynthesis and other forms of energy conversion and charge transduction.

**Supplementary Information:**

The online version contains supplementary material available at 10.1007/s11120-021-00824-4.

## Introduction

**Fig. 1:**
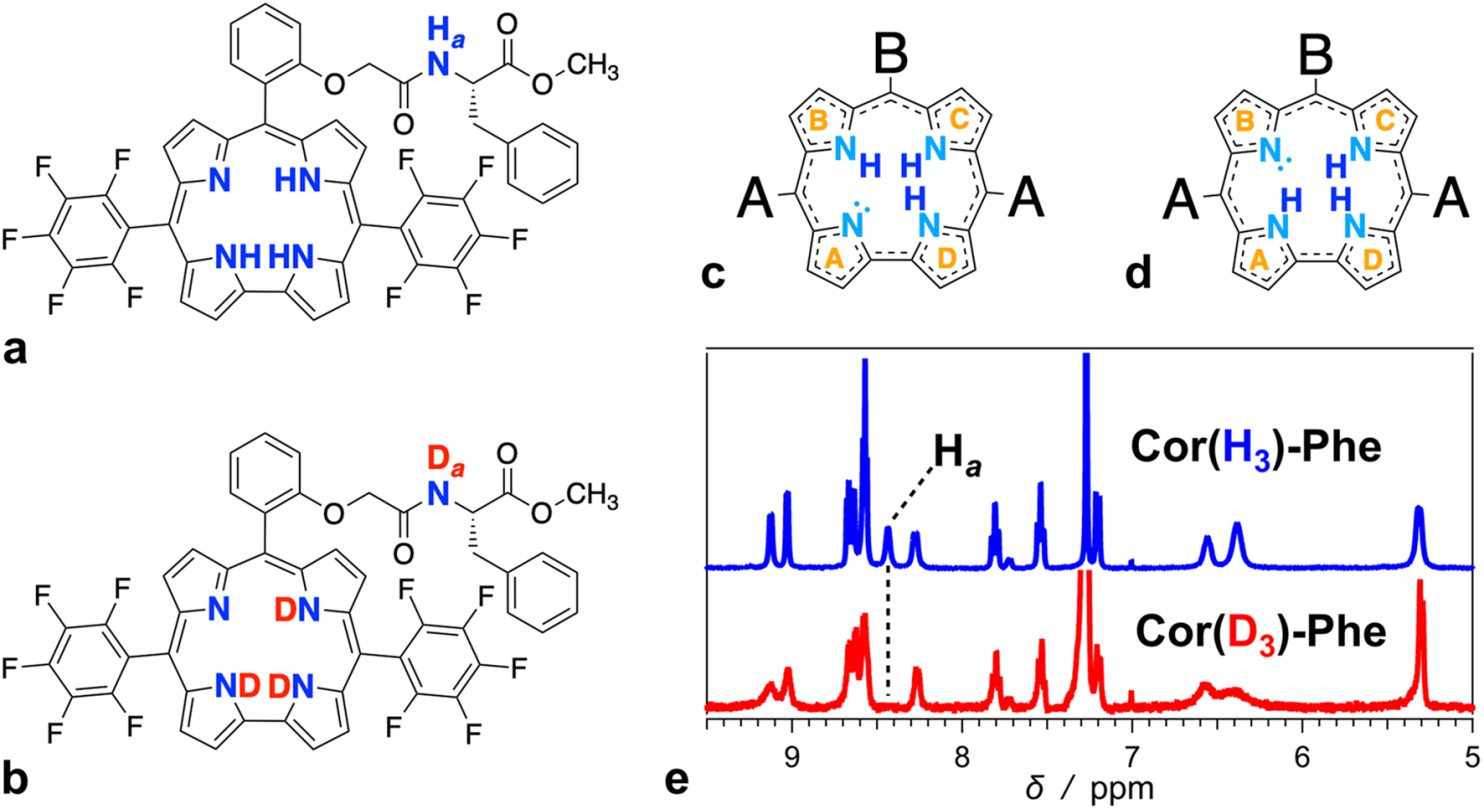
Structures of **a** **Cor(H**_**3**_**)-Phe**, **b** **Cor(D**_**3**_**)-Phe**, **c** tautomer 1 (T1), and **d** tautomer 2 (T2) along with **e** the aromatic region of the ^1^H NMR spectra (for CDCl_3_) of **Cor(H**_**3**_**)-Phe** and **Cor(D**_**3**_**)-Phe** (three washes of a **Cor(H**_**3**_**)-Phe** chloroform solution with D_2_O yield **Cor(D**_**3**_**)-Phe**, as the replacement of the amide proton, H_*a*_, indicates)

This article describes the excited-state dynamics and the optical properties of a free-base (FB) corrole modified with l-phenylalanine, **Cor(H**_**3**_**)-Phe** (Fig. 1). Deuteration of this conjugate does not noticeably affect its optical properties, such as absorption and emission spectra, as well as the excited-state lifetime. Picosecond transitions ascribed to excited-state tautomerization, on the other hand, manifest substantial kinetic isotope effects. While the tautomerization timescales are considerably faster than the deactivation to the ground state, they can be comparable to charge-transfer and energy-transfer reactions important for the design of systems for artificial photosynthesis.

As important as the discovery of corroles was in the 1960s (Johnson and Price 1960a, b; Johnson and Kay 1961, 1964, 1965), it was four decades later when the first reports of the one-pot syntheses of such macrocycles drew considerable attention to these chromophores by making them readily accessible (Gryko and Jadach 2001; Gross et al. 1999; Paolesse et al. 2001). Similar to porphyrins, corroles are tetrapyrroles with one less carbon in the macrocycle, resulting in electron-rich chromophores with properties that are complementary to those of other porphyrinoids (Gryko 2002; Orlowski et al. 2017a; Aviv-Harel and Gross 2009; Ghosh 2017; Rabinovich et al. 2011; Pomarico et al. 2018; Einrem et al. 2016). The importance of corroles for solar energy conversion, photocatalysis, photodynamic therapy and other areas of photonics is well recognized (Dogutan et al. 2011; Mahammed and Gross 2006; Agadjanian et al. 2009; Flamigni et al. 2008; Teo et al. 2017).

In addition to increasing the electron density, the contraction of the macrocycle lowers the molecular symmetry. While many porphyrins belong to *D*_4*h*_ and *D*_2*h*_ point groups, the symmetry of planar corroles cannot exceed *C*_2*v*_. Furthermore, the sterics and the tension in such contracted macrocycles force the pyrrole rings in the FB corroles out of plane leading to *C*_*s*_ and even *C*_1_ symmetry. Such displacement of the pyrrole N–H groups out of the macrocycle plane of the FB corroles drastically increases their acidity and propensity for hydrogen bonding (Szymanski et al. 2014). Even moderately polar solvents with weak basicity can deprotonate these FB porphyrinoids (Kruk et al. 2012).

The non-planar structure of FB corroles, exposing the pyrrole nitrogens to the media, enhances not only their acidity, but also their basicity. The p*K*_*a*_ values for non-protonated corroles, i.e., Cor(H_3_), range between 4 and 7, and monoprotonated ones, i.e., Cor(H_4_)^+^, exhibit p*K*_*a*_ in the range of 2–3 for aqueous media (Mahammed et al. 2003; Shen et al. 2007). They are quite smaller than the p*K*_*a*_ values for FB porphyrins, requiring strong acids to protonate them (p*K*_*a*_ ~ 4) and even stronger bases to deprotonate them (p*K*_*a*_ > 30) in aqueous/organic media (Jimenez et al. 1991).

That is, the macrocycle arrangements push the protonated, rather than the deprotonated, pyrrole nitrogens out of plane improving their accessibility. In fact, the basicity of FB corroles and porphyrins is similar, with their monoprotonated forms exhibiting similar p*K*_*a*_ values ranging between about 5 and 6 for aqueous media.

In their macrocycles, non-ionized FB corroles contain three protons distributed over four pyrrole nitrogens. Because of the molecular asymmetry, the four pyrroles are not equivalent. This feature results in discernable tautomers of FB corroles, as determined by the position of the deprotonated pyrrole nitrogen. FB corroles with *C*_*s*_ symmetry exist in the form of two tautomers. In the first tautomer, T1, the deprotonated nitrogen is on one of the two directly linked pyrroles, i.e., ring A or D (Fig. 1c). In the second tautomer, T2, one of the two pyrroles, linked to methines on both sides, i.e., ring B or C, is deprotonated (Fig. 1d). Lowering the molecular symmetry to *C*_1_ increases the number of possible tautomers of FB corroles.

In the ground state, the energy difference between T1 and T2 is small. It does not exceed 5 meV and T2 is slightly favored over T1 (Chandler et al. 2009; Kruk et al. 2012; Ding et al. 2005). This small energy difference results almost equimolar populations of the two tautomers at room temperature. In the excited states, on the other hand, the energy level of the tautomer with lesser crowding of the protons, i.e., T1, is about 100 meV below that of T2 (Szymanski et al. 2014; Kruk et al. 2012; Ding et al. 2005). These energy differences suggest that T1 defines the photophysics of FB corroles, assuming sufficiently fast tautomerization. Therefore, while the absorption spectra reveal transitions involving T1 and T2, the fluorescence spectra at room temperature (for non-polar liquid media) show solely the emission from T1 that is bathochromically shifted in comparison to where T2 emits (Szymanski et al. 2014; Kruk et al. 2012; Ding et al. 2005).

The differences in the electronic properties between the tautomers result not only in different acidities and photophysical characteristics, but also in different propensities of the FB corroles to act as electron and energy donors and acceptors. Photoinduced processes that are slower than the tautomerization rates should originate from the lowest excited state of the energetically preferred structure. Conversely, the starting points of photoinduced reactions occurring in timescales considerably shorter than the time constants of tautomerization, i.e., *τ* = *k*^–1^ ≪ *τ*_taut_, are representative of the ground-state population distribution of the different structures. This feature warrants the need for understanding the tautomerization dynamics of FB corrles in order to broaden and optimize their utility for photonics and biophotonics.

Herein, we focus on the excited-state dynamics of an FB *trans*-A_2_B-corrole linked with a phenylalanine, **Cor(H**_**3**_**)-Phe** (Fig. 1a). Transient-absorption (TA) spectroscopy reveals transitions in the 10 to 100 ps timescales, which are consistent with excited-state tautomerization and exhibit hydrogen kinetic isotope effects ranging between 2.6 and 3.9. These findings indicate that the dynamics of tautomerization of FB corroles does not affect nanosecond and slower processes, i.e., they originate from equilibrium excited-state populations of tautomers. The excited-state tautomerization dynamics, however, can be deterministic for femtosecond and picosecond reactions, such as charge injection from photoexcited FB corroles into semiconductors, essential for light harvesting, energy conversion, and overall for artificial photosynthesis.

## Results and discussion

### Corrole design

**Scheme 1:**
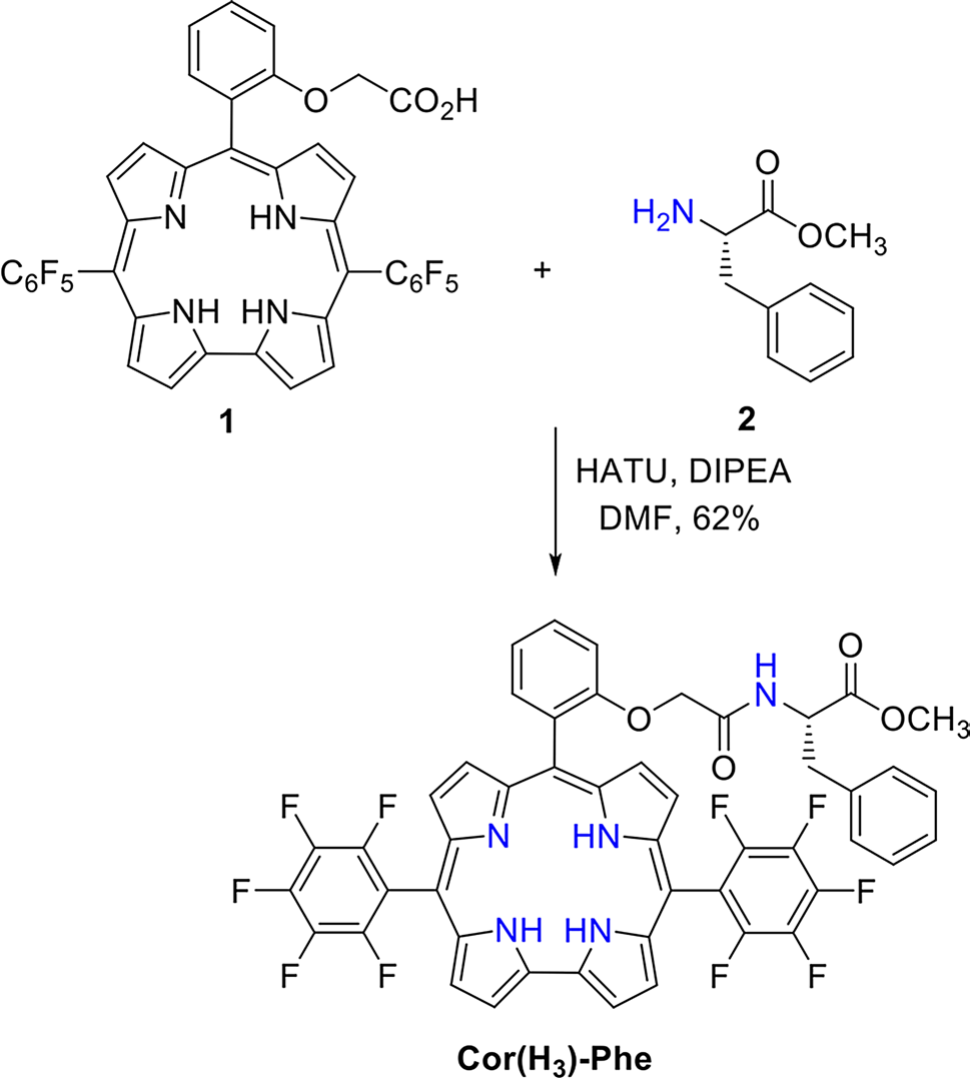
Synthesis of **Cor(H**_**3**_**)-Phe**

Corroles, containing *O*-linked glycolamides to the *ortho* position of *meso*-phenyl groups, exhibit propensity for intramolecular hydrogen bonding important for the design of supramolecular structures with tight conformations (Orlowski et al. 2017b). Therefore, we select an FB *trans*-A_2_B-corrole with an *o*-C_6_H_4_-OCH_2_CONH-R *meso*-substituent containing l-phenylalanine, i.e., -R = -C^(S)^H(-CH_2_-C_6_H_5_)-CO_2_CH_3_., i.e., **Cor(H**_**3**_**)-Phe** (Scheme 1). Linking these porphyrinoids to amino acids and peptides offers building blocks for biomimetic and bioinspired charge-transfer systems (Vullev 2011). Following a classical procedure for peptide synthesis allows us to prepare **Cor(H**_**3**_**)-Phe** from corrole **1** and l-phenylalanine methyl ester (**2**) in 62% yield after purifying with column chromatography (Scheme 1).

Multiple treatments of **Cor(H**_**3**_**)-Phe** dissolved in a chlorinated solvent with D_2_O drives the exchange of the amide, H_*a*_, and the three pyrrole N–H protons with deuterium to obtain **Cor(D**_**3**_**)-Phe** (Fig. 1a, b). NMR spectroscopy allows for monitoring the replacement of these protons with deuteriums. After treatments with D_2_O, and prior to the optical measurements, we acquire the ^1^H NMR spectra of the samples in a volatile deuterated solvent, such as CDCl_3_, to confirm the proton-to-deuterium exchange. Fast evaporation of the volatile CDCl_3_ in vacuo at room temperature leaves the solid **Cor(D**_**3**_**)-Phe** that we dissolve in a spectroscopy-grade non-polar solvent. With N–H p*K*_*a*_ < 7 (Ghosh 2017), the pyrrole protons are quite labile and not always detectable in an NMR solvent even with moderate polarity. Also, these protons are in the middle of the ring current, induced in the corrole macrocycle, and they exhibit pronounced shielding effects with chemical shifts between 0 and  − 6 ppm for toluene-*d*_8_ (Bocian et al. 2015). Nevertheless, we monitor the amide proton, H_*a*_, in **Cor(H**_**3**_**)-Phe** (Fig. 1a), which disappear from the ^1^H NMR spectrum upon deuterium exchange (Fig. 1e). Because amide protons are less labile than the pyrrole N–H protons, the replacement of H_*a*_ with D_*a*_ (Fig. 1a, b) ensures that the pyrrole N–H protons are also replaced with deuteriums.

The relatively low p*K*_*a*_ of the pyrrole N–H groups further decreases upon photoexcitation. In order to prevent deprotonation of the corrole pyrroles, especially when in excited state, this pronounced acidity renders the use of polar solvents, along with non-polar Lewis bases, unfeasible. Furthermore, FB corroles have a propensity for photodegradation in polar media, such as acetonitrile, as the teams of Gryko and Danikiewicz demonstrate using mass spectrometry (Swider et al. 2010). Selection of an aromatic non-polar solvent, such as toluene, ensures sufficient solubility of the corrole conjugates while suppressing the deprotonation and photodegradation of these contracted FB porphyrinoids.

### Optical properties

**Fig. 2:**
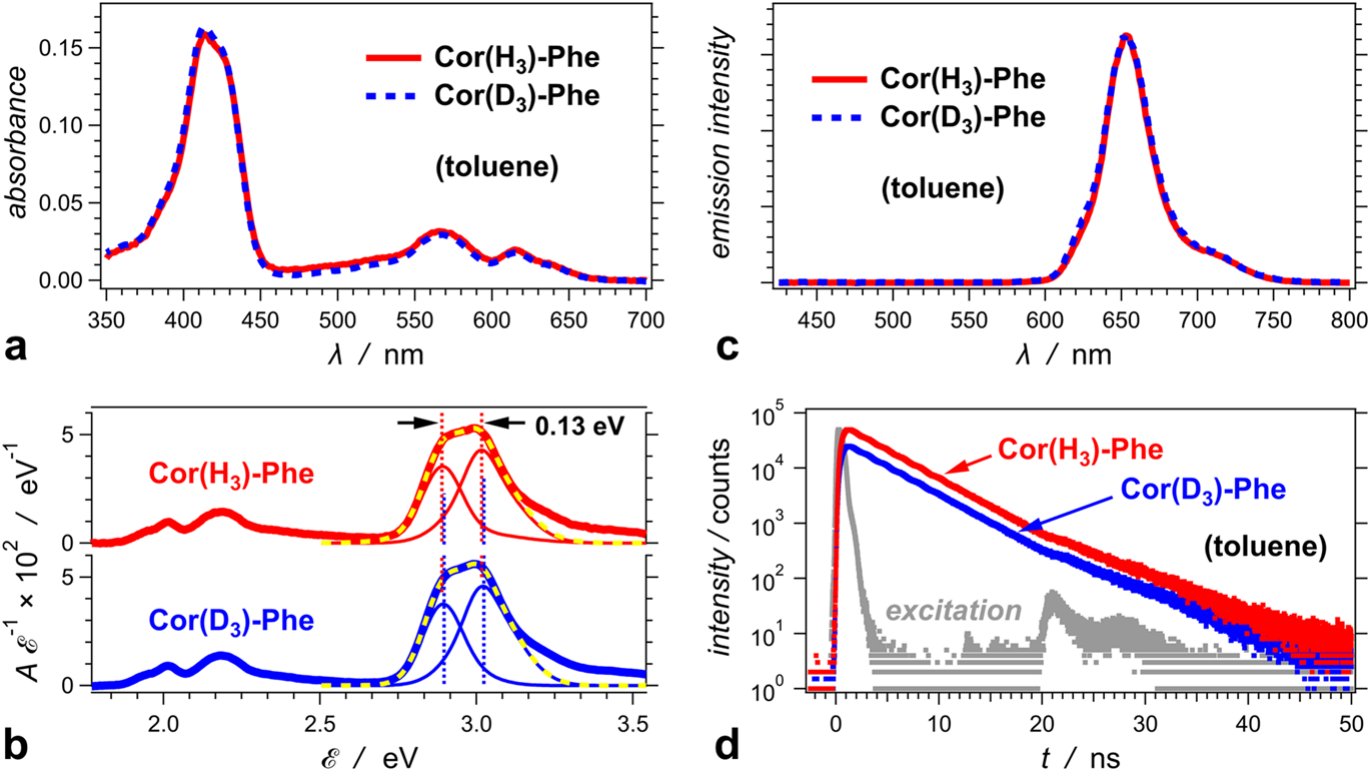
Optical absorption and emission properties of **Cor(H**_**3**_**)-Phe** and **Cor(D**_**3**_**)-Phe** for toluene media. **a** Optical absorption spectra of 1.5 μM corrole samples. **b** The optical absorption spectra plotted against energy abscissa. The recorded *A*(*λ*) spectra are converted to A(E) as previously described (Angulo et al. 2006). The Soret band at 3 eV is deconvoluted by fitting it to a sum of Gaussians. The yellow dashed lines represent the data fits, and the thin lines show the comprising components of the band. **c** Fluorescence spectra of the protonated and the deuterated corrole conjugate; *λ*_*ex*_ = 415 nm; and ϕ_*f*_ of **Cor(H**_**3**_**)-Phe** and **Cor(D**_**3**_**)-Phe** is 0.15 and 0.13, respectively. **d** Fluorescence decays of the protonated and the deuterated corrole conjugate recorded using TCSPC; *λ*_*ex*_ = 406 nm; half-height excitation pulse width = 200 ps; and *τ* of **Cor(H**_**3**_**)-Phe** and **Cor(D**_**3**_**)-Phe** is 4.2 ns and 4.3 ns, respectively. For improved visualization, the ordinate for the emission decay of **Cor(H**_**3**_**)-Phe** is set from 1 to 10^5^ counts, and for **Cor(D**_**3**_**)-Phe**—from 2 to 2 × 10^5^ counts. The secondary excitation pulse at 20–30 ns is an instrumental artifact, most likely originating from ringing due to an impedance mismatch in circuits and electric connections. Nevertheless, the intensity of this secondary excitation pulse is about 0.1% of the intensity of the initial excitation and does not considerably affect the decay curves. Furthermore, the deconvolution algorithm accounts for this weak secondary excitation pulse and does not affect the extracted lifetimes from the measured decays

Typical for porphyrinoids, the optical absorption spectrum of **Cor(H**_**3**_**)-Phe** shows a Soret band stretching between about 380 and 440 nm, and Q-bands spreading between 500 and 650 nm (Fig. 2a). The shape of the Soret band indicates that it comprises two principal peaks. Spectral deconvolution reveals that these two components are separated by about 0.13 eV (Fig. 2b), which can be ascribed to S_0_ → S_2_ transitions of two tautomers (Szymanski et al. 2014; Kruk et al. 2012; Ding et al. 2005).

The fluorescence of **Cor(H**_**3**_**)-Phe** shows a spectral band consistent with the radiative deactivation of T1 (Fig. 2c) (Szymanski et al. 2014; Kruk et al. 2012; Ventura et al. 2005; Ding et al. 2005). The modest fluorescence quantum yields, ϕ_*f*_ = 0.15, along with the nanosecond excited-state lifetime, *τ* = 4.2 ns (Fig. 2d), reveals non-radiative decay rates, *k*_*nr*_ = 2 × 10^8^ s^–1^, that are about six times larger than the radiative ones.

Replacing the pyrrole protons with deuteriums does not noticeably affect the steady-state optical spectra of the corrole conjugate (Fig. 2a–c). That is, deuteration does not noticeably alter the distribution of the ground-state tautomer populations in non-polar liquid media at room temperature. As estimated from emission decays recorded using time-correlated single-photon counting (TCSPC), the excited-state lifetimes of **Cor(H**_**3**_**)-Phe** and **Cor(D**_**3**_**)-Phe** are practically the same (Fig. 2d). This lack of detectable kinetic isotope effects indicate that the N–H vibrational modes in **Cor(H**_**3**_**)-Phe** are not deterministic for the deactivation of its lowest singlet excited state. The lack of isotope effects on the measured nanosecond dynamics at room temperature, however, is not necessarily informative about the picosecond excited-state transformations.

### Transient-absorption dynamics

**Fig. 3:**
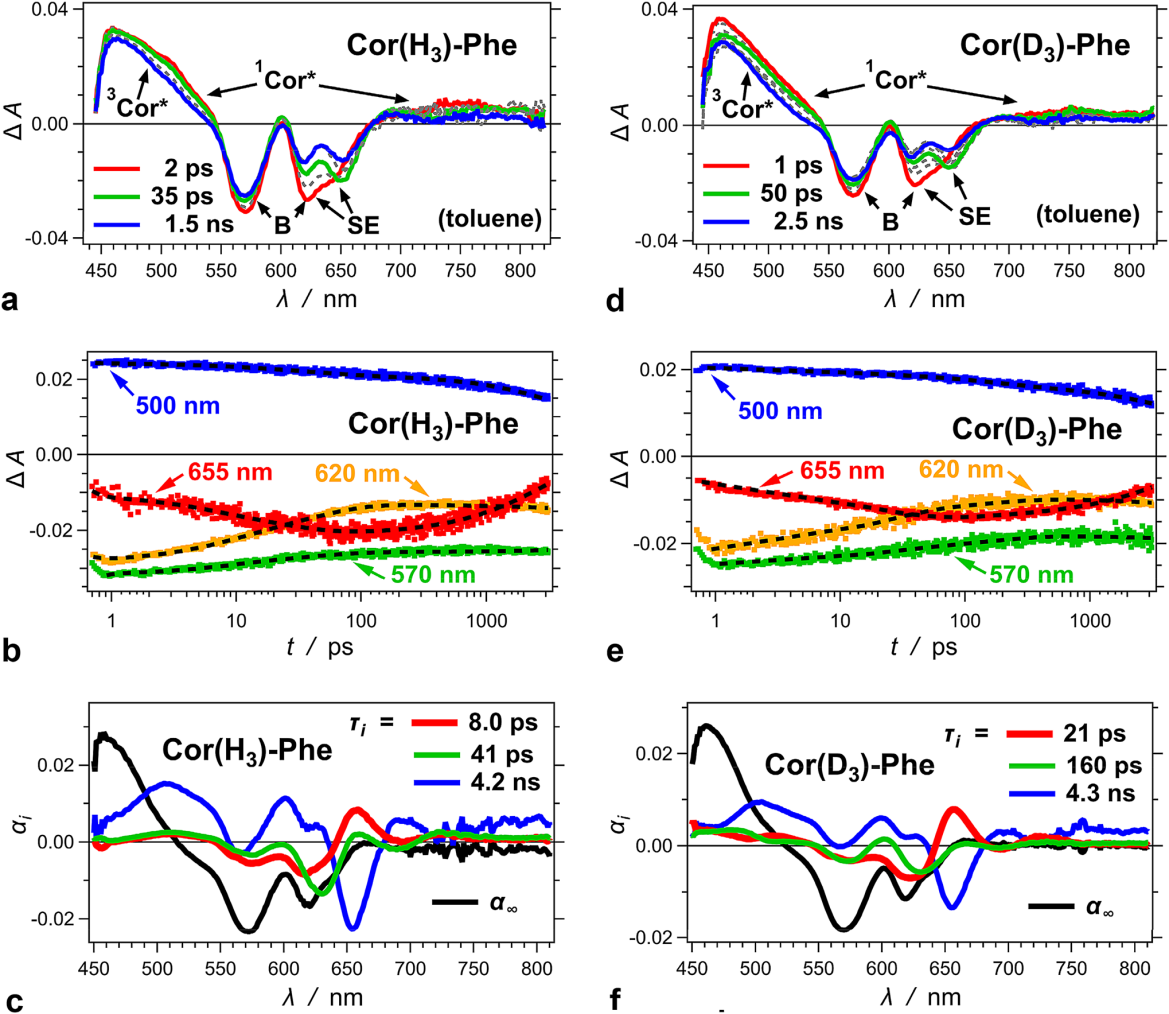
Transient-absorption dynamics of **Cor(H**_**3**_**)-Phe** and **Cor(D**_**3**_**)-Phe** for toluene media; *λ*_*ex*_ = 400 nm; pulse width = 50 fs. **a**, **d** TA spectra showing the absorption features of ^1^Cor* and ^3^Cor*, as well as the ground-state bleach (B) and the stimulated emission (SE). For clarity, only the spectra at picosecond (1–2 ps), tens of picosecond (35–50 ps), and nanosecond (1–3 ns) times are displayed in color. Additional TA spectra at intermediate timepoints are depicted in the background with gray dashed lines. **b**, **e** TA kinetic traces recorded at 500 nm, where ^1^Cor* and ^3^Cor* absorb; 570 nm, where the B is prevalent and has some overlap with the TA of ^1^Cor*; 620 nm, where B and SE overlap; and 655 nm, where SE corresponds to the fluorescence maximum. The dashed lines represent the global fits. **c**, **f** Amplitude spectra obtained from global-fit analysis (Eq. 1), aided by single value decomposition, showing the picosecond and nanosecond transitions, as well as the long-lived ^3^Cor* along with the B depicted by *α*_∞_ vs. *λ*. The nanosecond time constants, i.e., 4.2 and 4.3 ns, are obtained from the fluorescence decays (Fig. 2d) and introduced to the global-fit algorithm with their values held

Optical TA spectra of **Cor(H**_**3**_**)-Phe** reveal a broad absorption band stretching between 450 and 550 nm (Fig. 3a). It is typical for the overlapping absorptions of the singlet and triplet excited-state transients of corroles (Flamigni et al. 2008; Flamigni and Gryko 2009; Tasior et al. 2008). In addition, the singlet transient, ^1^Cor*, manifests weak broad TA in the near-infrared (NIR) spectral region (Fig. 3a). The negative Δ*A* peaks are consistent with the ground-state bleach (B) and the stimulated emission (SE) of corroles (Fig. 3a). The 650-nm SE peak matches the steady-state fluorescence that originates from the T1 tautomer (Fig. 2c). Nevertheless, the ^1^T2* tautomer exhibits hypsochromically shifted emission (Kruk et al. 2012) that is most plausibly responsible for an SE signal at the early timepoint around 620 nm (Fig. 3a). The bleach, B, at 570 nm undergoes about 13% recovery within the first 35 ps after the excitation. At 620 nm, on the other hand, the negative amplitude of the signal shows a 34% decrease within the first 35 ps (Fig. 3a). This discrepancy precludes that the bleach recovery by itself is solely responsible for the early dynamics of the signal around 620 nm and renders a principal contribution of the SE from the ^1^T2* excited state as a plausibility.

The TA dynamics is consistent with the nanosecond deactivation of ^1^Cor*, as the bleach recovery and the TA decays in the blue and NIR spectral regions reveal (Fig. 3b). The lack of significant decrease in Δ*A* of the 450–550-nm TA band suggests for comparable molar extinction coefficients of ^1^Cor* and ^3^Cor* in this spectral region, along with a relatively high quantum yield of intersystem crossing (ISC), ϕ_ISC_. The nanosecond magnitude of *k*_*nr*_^–1^ precludes the option for femtosecond and picosecond ISC observed for other triplet-forming chromophores (Zugazagoitia et al. 2008; Espinoza et al. 2016).

The SE bands reveal the most interesting feature of the picosecond TA dynamics. At the fluorescence maximum, i.e., at 655 nm, the intensity of SE shows a picosecond increase, followed by a nanosecond decrease (Fig. 3b). Concurrently, the bands at 620 nm and 570 nm show a decrease in –Δ*A* with the same picosecond time scales. These changes are consistent with ^1^T2* → ^1^T1* tautomerization. The growth of the SE fluorescence band associates with T1 and accompanies a drop of hypsochromically shifted SE ascribed to T2.

The behavior of the –Δ*A* bands at 570 and 620 nm at *t* > 100 ps is consistent with an overlap of the ground-state bleach with the TA of ^1^Cor*. The nanosecond deactivation of ^1^Cor* proceeds along three parallel pathways: (1) a radiative decay (F); (2) a non-radiative decay via internal conversion (IC); and (3) another non-radiative route, via ISC. While all these pathways lead to a decrease in the ^1^Cor* TA, i.e., making Δ*A* more negative, only F and IC lead to recovery of the ground state and making Δ*A* more positive. Therefore, the balance between the absorptivity of ^1^Cor and ^1^Cor*, along with the magnitude of ϕ_ISC_, determines weather Δ*A* at these wavelengths exhibits positive or negative nanosecond shifts or just remains constant.

To quantify the observed picosecond excited-state dynamics, we resort to global-fit analysis of the TA results (Fig. 3c):

1 $$ \Delta A\left( {\lambda ,t} \right) = \alpha_{\infty } \left( \lambda \right) + \Sigma_{i} \alpha_{i} \left( \lambda \right){\text{exp}}\left( {{-}t\tau_{i}^{{{-}{1}}} } \right) $$

where we hold the time constants, *τ*_*i*_, which is the same for different wavelengths. A positive amplitude, i.e., *α*_*i*_(*λ*) > 0, indicates a negative Δ*A* shift at that wavelength, which is consistent with TA decay or a growth of SE or B signals. Conversely, a negative amplitude shows a positive Δ*A* shift, i.e., a TA rise or a decrease in the intensity of SE or B signals. In addition, *α*_∞_(*λ*) ≠ 0 is suggested for long-lived transients with decays that are considerably slower than the dynamic range of the pump–probe technique.

The amplitude spectra show biexponential picosecond dynamics consistent with a growth of SE at 655 nm as a concurrent decrease in hypsochromic emission signal at about 620 nm (Fig. 3c). Small changes in the bleach at about 570 nm and the TA of ^1^Cor* can be ascribed to spectral shifts. In a good agreement with tautomerization, these results show heterogeneous kinetics with timescales of about 10–40 ps (Fig. 3c, Table 1).

**Table 1:** Rate constants of the picosecond and nanosecond excited-state dynamics of **Cor(H**_**3**_**)-Phe** and **Cor(D**_**3**_**)-Phe**

	*k*_i_* × 10^–9^/s^–1^ (*α*_*i*_) ^a^	KIE_i_* ^b^	*k*_*d*_ × 10^–9^/s^–1^ ^c^	KIE_*d*_^b^
Cor(H_3_)-Phe	130 ± 20 (0.44) 24 ± 1 (0.66)		0.24 ± 0.01	
Cor(D_3_)-Phe	48 ± 3 (0.63) 6.3 ± 0.5 (0.47)	2.7 ± 0.4 3.8 ± 0.3	0.23 ± 0.01	1.04 ± 0.06

^a^Rate constants representing the picosecond dynamics dominated by ^1^T2* ⟶ ^1^T1* transitions, along with the relative amplitudes in the parentheses, i.e., *α*_*i*_ = Δ*A*_*i*,(620 nm, 0 ps)_*/*Σ_*j*_ Δ*A*_*j*,(620 nm, 0 ps)_

^b^Kinetic isotope effects obtained from comparison of the rate constants for **Cor(H**_**3**_**)-Phe** (*k*^(H)^) and **Cor(D**_**3**_**)-Phe** (*k*^(D)^), i.e., KIE_*i*_ = *k*_*i*_^(H)^/*k*_*i*_^(D)^. The error bars, *δ*KIE, are obtained from the error bars for the rate constants, *δk*, i.e., *δ*KIE_*i*_ = KIE_*i*_ ((*δk*_*i*_^(H)^*/k*_*i*_^(H)^)^2^ + (*δk*_*i*_^(D)^*/k*_*i*_^(D)^)^2^)^–2^

^c^Rate constants for the deactivation of the S_1_ states are obtained from the fluorescence decays of the two conjugates (Fig. 2d)

The heterogeneity of the kinetics representing this excited-state transformation reveals another important feature of **Cor(H**_**3**_**)-Phe**. For clarity of the presentation, in this publication, we describe the excited-state transitions in terms of two tautomers, T1 and T2, as previously described for corroles with *C*_*s*_ symmetry (Kruk et al. 2012; Szymanski et al. 2014; Ding et al. 2005). Hydrogen bonding between the glycolamide substituent and the pyrroles, however, can further break the symmetry and increase the number of discernable tautomeric structures. Excluding its hypsochromic shoulder, the Soret ground-state absorption band shows at least two principle components (Fig. 2b). Also, we cannot rule the possibility of different tautomers with closely similar energies of their S_0_ → S_*n*_ transitions. In addition, the emission spectrum of **Cor(H**_**3**_**)-Phe** unequivocally shows the features of the T1 fluorescence. Therefore, the two-tautomer model provides a good approximation for describing the optical properties of **Cor(H**_**3**_**)-Phe**. The heterogeneity of the picosecond dynamics, however, points to a removal of degeneracy that intramolecular hydrogen bonding induces in **Cor(H**_**3**_**)-Phe**. Placing three protons on four pyrrole nitrogens defines two degenerate structures of T1 where the deprotonation of pyrrole A and pyrrole D is equivalent (Fig. 1; and two degenerate structures of T2, i.e., with equivalence of deprotonated pyrroles B and C (Fig. 1). Intramolecular hydrogen bonding between the glycolamide and one of the pyrroles makes the two structures of T2 (and T1) different.

### Kinetic isotope effects

The TA spectra of the deuterated conjugate, **Cor(D**_**3**_**)-Phe**, show the same absorption, B, and SE feature as those of **Cor(H**_**3**_**)-Phe** (Fig. 3d). While the overall TA kinetic traces (Fig. 3b, e) and global-fit amplitude spectra (Fig. 3c, f) appear similar for the protonated and deuterated conjugates, the principal differences are in the picosecond dynamics.

The trace at 655 nm, ascribed to the T1 fluorescence, shows faster SE growth for **Cor(H**_**3**_**)-Phe** than for **Cor(D**_**3**_**)-Phe** (Fig. 3c, f). Similarly, the deuteration slows down the picosecond dynamics depicted at 620 nm. The global-fit analysis reveals biexponential picosecond kinetics for **Cor(D**_**3**_**)-Phe** that is about three to four times slower than that for **Cor(H**_**3**_**)-Phe** (Fig. 3c, f).

The kinetic isotope effect (KIE) when comparing the rate constants of the fast components of the picosecond dynamics of **Cor(H**_**3**_**)-Phe** and **Cor(D**_**3**_**)-Phe** amounts to 2.7. Similarly, for the slow components, KIE = 3.8 (Table 1). The magnitude of these KIE values indicate that the proton vibrational modes have direct contributions to the transitions observed in the picosecond time domain, which is consistent with ascribing them to excited-state tautomerization.

Despite the large KIE for the picosecond dynamics, we do not detect KIE in the nanosecond deactivation of the emissive excited state (Fig. 2d), i.e., KIE < 1.05 (Table 1). This outcome indicates that the picosecond tautomerization relaxes fast enough the excited states to ^1^T1* type of a structure, which is solely responsible for the basic photophysics of **Cor(H**_**3**_**)-Phe**. Under the employed conditions, the direct deactivation of high-energy tautomers to the ground state does not have detectible contributions to the observed nanosecond photophysics, which is consistent with Kasha’s rule (Kasha 1950).

## Conclusion

The foundation of the Kasha’s rule originates from the common trend that the coupling between electronically excited states tends to be inherently stronger than their coupling with the ground state. Hence, the relaxation of upper excited states to the lowest excited one, within the same multiplicity manifold, is much faster than the direct transitions from them to the ground state. In organic chromophores, therefore, most photoinduced reactions commence from the lowest singlet excited state. Femtosecond processes from upper excited states, important for generating hot electrons and holes, for example, can successfully compete with the internal conversion to the lowest excited state. Free-base corroles reveal a slightly different paradigm. The picosecond excited-state tautomerization, responsible for relaxation of about 0.1 eV, is fast enough so that the nanosecond deactivation processes occur from the structure with the lowest energy in the singlet manifold. Nevertheless, the proton transfer involved in the tautomerization is most often inherently slower than internal conversion between electronically excited states. Therefore, picosecond and sub-picosecond charge and energy transfer can readily occur from high-energy tautomers benefiting from the extra 100 meV thermodynamic driving forces. This characteristic of free-base corroles can be extended to other porphyrinoids (Thomas et al. 2020) and illustrates alternative paradigms in the design of artificial photosynthesis with broad applicability to a range of chromophores exhibiting such excited-state behavior.

## Materials and methods

### Synthesis

Reaction was carried out in flame-dried glassware in argon atmosphere. Required chemicals, including l-phenylalanine methyl ester (**2**), were purchased from Sigma-Aldrich and used without further purification. DMF was dried using solvent purification system. Corrole **1** was prepared according to a reported procedure (Orlowski et al. 2015). The reaction progress was monitored by thin layer chromatography (TLC, aluminum plates coated with silica gel, Merck 60, F-254) and visualized via UV lamp. The ^1^H NMR spectrum was measured at temperature 298 K in CDCl_3_ (if not otherwise stated) solutions with a Varian vnmrs-600, using tetramethylsilane (TMS) as internal standard.

#### Cor(H_3_)-Phe (Scheme 1)

**1** (50 mg, 0.064 mmol), HATU (24 mg, 0.064 mmol), and DIPEA (17 µL, 0.096 mmol) were dissolved in dry DMF (18 mL) and stirred for 30 min under argon atmosphere. Subsequently, l-phenylalanine methyl ester (**2**) (0.064 mmol) was added and the resulting solution was stirred for 2 h. The crude reaction mixture was purified by column chromatography (silica, 1% methanol in DCM) and recrystallized from diethyl ether to produce 37 mg of dark red crystals of **Cor(H**_**3**_**)-Phe** (62% yield). ^1^H NMR (600 MHz, DMF-d_6_) *δ* (ppm): 9.36–9.17 (m, 2H), 9.13–8.54 (m, 4H), 8.72–8.53 (m, 2H), 8.14–8.07 (m, 1H), 7.85–7.76 (m, 1H), 7.52–7.45 (m, 1H), 7.44–7.37 (m, 1H), 6.72–6.04 (m, 4H), 5.99–5.57 (m, 2H), 4.58–4.36 (m, 2H), 4.06–3.96 (m, 1H), 3.29–3.03 (bs, 3H), 2.17–1.97 (m, 1H), and 1.90–1.32 (m, 1H). HRMS (ESI/TOF) *m/z*: [M – H]^–^ Calcd. for C_49_H_28_F_10_N_5_O_4_^–^ 940.1982; Found 940.1959.

#### Cor(D_3_)-Phe

1 mg of **Cor(H**_**3**_**)-Phe** was dissolved in 1 mL CDCl_3_ and its ^1^H NMR spectrum was recorded. Addition of 0.5 mL D_2_O was followed by shaking the mixture for 5 min, and after the phases were allowed to separate, the aqueous layer was pipetted out. The procedure was repeated three times, the proton-to-deuterium exchange was confirmed using NMR spectroscopy (Fig. 1e), the solvent was evaporated in vacuo, and the obtained dry **Cor(D**_**3**_**)-Phe** was dissolved in toluene for spectroscopy studies. For the solvent, using toluene dried over N_2_SO_4_, and toluene that was washed several times with D_2_O, does not result in detectable differences in the transient-absorption spectroscopy.

### Steady-state optical spectroscopy

The absorbance spectra were recorded using a JASCO V-670 UV/Visible/NIR spectrophotometer (Tokyo, Japan). Fluorescence measurements were conducted with a Horiba Jobin Yvon Fluorolog-3-22 spectrofluorometer (NJ, USA) (Lu et al. 2010; Chau et al. 2011; Gupta et al. 2013). All samples were purged with argon prior to each measurement. The absorbance at the excitation wavelengths was kept within the range between 0.1 and 0.2 for recording the spectra used for calculating the emission quantum yields (Demas and Crosby 1971; Wan et al. 2008; Jung et al. 2014; Jones et al. 1995).

### Time-resolved optical emission spectroscopy

Time-correlated single-photon counting (TCSPC) experiments were conducted using Horiba Jobin Yvon Fluorolog-3-22 spectrofluorometer (Tokyo, Japan)). Pulsed laser diode, NanoLED, provided the excitation at 406 nm at a repetition frequency of 1 MHz and half-height pule width of 200 ps (Krzeszewski et al. 2018; Ghazinejad et al. 2012; Espinoza et al. 2018). Data fits of the emission decays, employing deconvolution with a monoexponential function, yielded the singlet excited-state lifetimes.

### Transient-absorption spectroscopy

A Helios pump-probe spectrometer (Ultrafast Systems, LLC, Florida, USA) was used in a transmission mode. 800-nm pulses (≥ 35 fs, 4.0 mJ per pulse, at 1 kHz) were generated by a SpitFire Pro 35F regenerative amplifier (Spectra Physics, Newport, CA, USA). The amplifier was pumped with an Empower 30 Q-switched laser ran at 20 W. A MaiTai SP oscillator provided the seed pulses with 55-nm bandwidth. The wavelength of the pump was tuned using an optical parametric amplifier, OPA-800CU (Newport Corporation, Newport, CA, USA), equipped with harmonic generators. Responses from pure solvents were used for the chirp correction of the transient-absorption data. The data analysis was carried out using Surface Xplorer (Ultrafast Systems, LLC, Florida, USA) and IgorPro v. 8 (WaveMetrics, Inc., Lake Oswego, OR, USA) (Purc et al. 2016; Bao et al. 2014; Upadhyayula et al. 2015; Gray et al. 2017; Guo et al. 2013).

## Supplementary Information

Below is the link to the electronic supplementary material.

Supplementary file1 (PDF 141 KB)
