# Supplementary material for: How does tautomerization affect the excited-state dynamics of an amino acid-derivatized corrole?
Source: Photosynth Res. 2021 Mar 12;148(1-2):67–76. doi: 10.1007/s11120-021-00824-4 (PMC8154756; doi:10.1007/s11120-021-00824-4)
Supplement: Supplementary file 1 — Supplementary file1 (PDF 141 KB) [file 11120_2021_824_MOESM1_ESM.pdf]

Electronic Supplementary Material for:

How does tautomerization affect the excited-state dynamics of an amino acid-derivatized corrole?

John A. Clark[1,=] ▪ Rafał Orłowski[2,=] ▪ James B. Derr[3] ▪ Eli M. Espinoza[4,#] ▪ Daniel T. Gryko[2,*] ▪ Valentine I. Vullev[1,3,4,5,*]

[1] Department of Bioengineering, University of California, Riverside, CA 92521, U.S.A.
[2] Institute of Organic Chemistry, Polish Academy of Sciences, Kasprzaka 44/52, 01-224 Warsaw, Poland.
[3] Department of Biochemistry, University of California, Riverside, CA 92521, U.S.A.
[4] Department of Chemistry, University of California, Riverside, CA 92521, U.S.A.
[5] Materials Science and Engineering Program, University of California, Riverside, CA 92521, U.S.A.

[=] Equal contributions
[#] Present address: College of Bioengineering, University of California, Berkeley, CA 94720, U.S.A.

* vullev@ucr.edu, dtgryko@icho.edu.pl

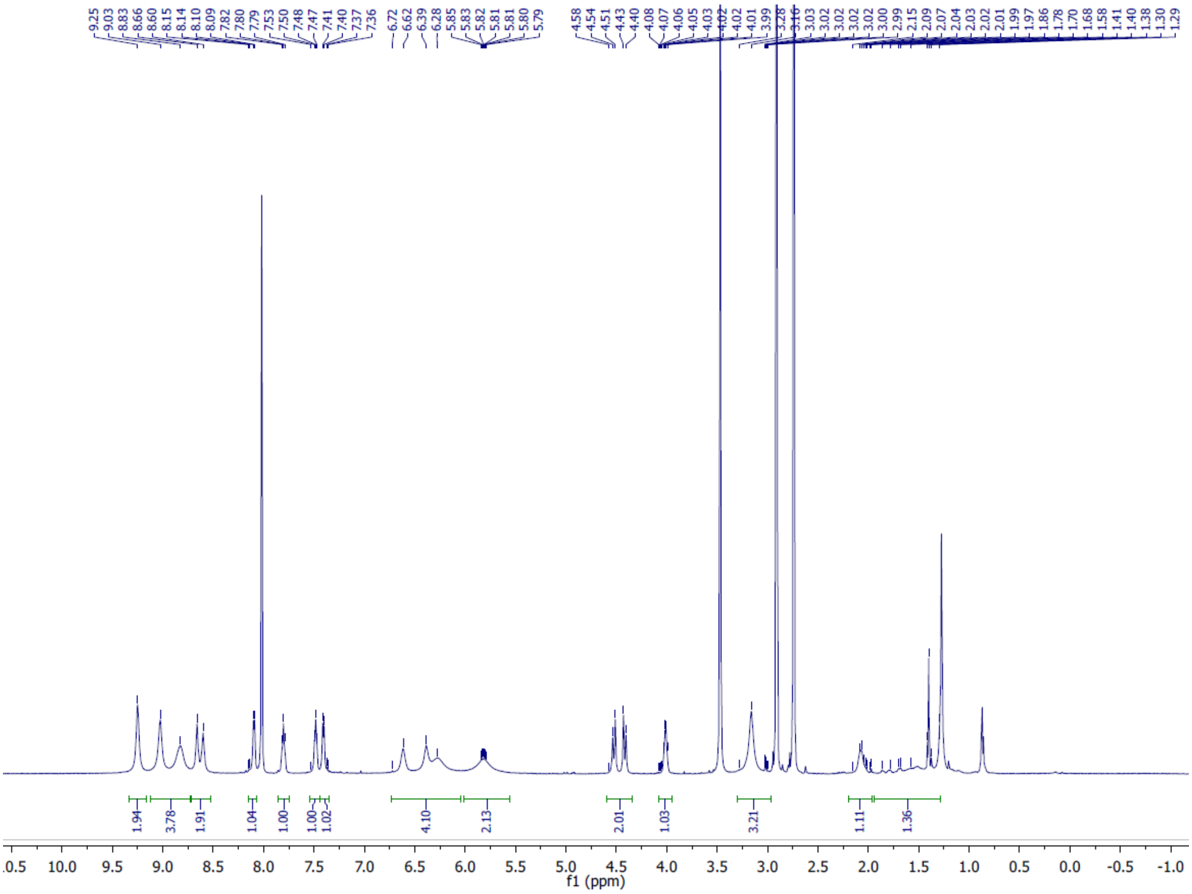

a

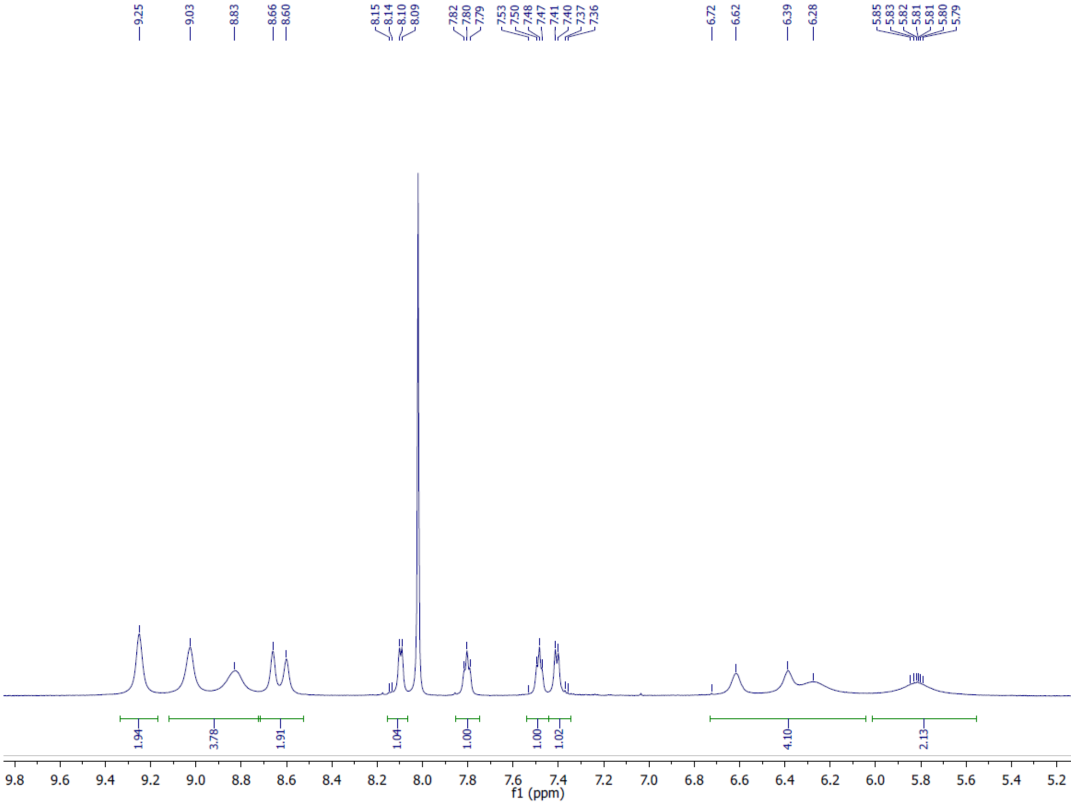

b

Figure S1. ^{1}H NMR spectra of **Cor(H3)-Phe** for DMF-d_5: (a) the whole spectral region; and (b) the aromatic region.
